# Methanogen Community Dynamics and Methanogenic Function Response to Solid Waste Decomposition

**DOI:** 10.3389/fmicb.2021.743827

**Published:** 2021-10-11

**Authors:** Shu Yang, Lei Li, Xuya Peng, Rui Zhang, Liyan Song

**Affiliations:** ^1^Key Laboratory of Three Gorges Reservoir Region’s Eco-Environment, Ministry of Education, Chongqing University, Chongqing, China; ^2^Environmental Microbiology and Ecology Research Center, Chongqing Institute of Green and Intelligent Technology, Chinese Academy of Sciences, Chongqing, China; ^3^School of Resources and Environmental Engineering, Anhui University, Hefei, China

**Keywords:** solid waste decomposition, methanogen community composition, methanogenic pathways, dynamics, mechanism

## Abstract

Methane production during solid waste decomposition is a typical methanogen-mediated and enzyme-catalyzed anaerobic digestion (AD). Methanogen community dynamics and metabolic diversity during the decomposition are not known. In this study, we investigated methanogen community dynamics and methanogenic pathways during solid waste decomposition in a bioreactor using high-throughput Illumina MiSeq sequencing and phylogenetic investigation of communities by reconstruction of unobserved states (PICRUSt), respectively. We also related the methanogen community differences with solid waste and leachate physiochemical parameters. Results showed that the percentage of biodegradable matter (BDM) in solid waste decreased from 55 ± 5% in aerobic phase (AP) to 30 ± 2% in anaerobic acid phase (ACP), and to 13 ± 11% in methanogenic phase (MP), resulting in 76% BDM consumption by microbes. Methanogen community structure varied in AP, ACP, and MP, showing that *Methanomicrobiales* and *Methanosarcinales* were dominant in AP and MP and archaea E2 was abundant in ACP. Each phase had abundant core methanogen orders, genera, and species with significant difference (*p* < 0.05). Redundancy analysis showed that biochemical oxygen demand (BOD_5_) and nitrate significantly influenced methanogen community composition, suggesting that methanogen community structure is nutrient-dependent. Two methanogenic pathways including acetoclastic and hydrogenotrophic pathways with associated functional genes differed at three phases. ACP had the lowest abundance of these genes, indicating that methanogenesis was inhibited in acidogenesis. Abundant hydrogenotrophic and acetoclastic methanogenesis functional genes in MP and AP are in response to the abundance of *Methanomicrobiales* and *Methanosarcinales*. The findings provide previously unidentified insight into the mechanism of methanogen community structure and function during solid waste bioconversion for methane.

## Graphical Abstract

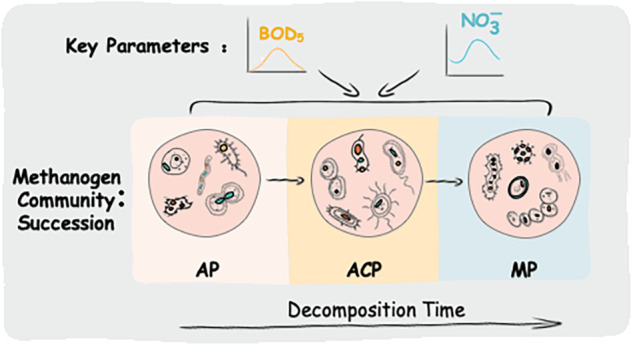

## Introduction

The world is facing the challenges of energy shortage and climate change. Various innovation techniques are developing to produce and recover clean energy such as methane (Niu et al., 2014; Li et al., 2017b). Municipal solid waste (MSW) landfills are an important methane resource, representing 22% of global anthropogenic emissions (IPCC, 2013). Recently, Powell et al. (2016) investigated 850 landfills in the United States and found that the CH_4_ emissions from landfills are largely underestimated (262 million tons vs. 121 million tons estimated by United States EPA), suggesting that more bioenergy is stored in landfills.

Methane production (methanogenesis) in solid waste decomposition is a typical methanogen-mediated and enzyme-catalyzed anaerobic digestion. Methanogenesis uses carbon such as acetic acid and carbon dioxide as the terminal electron acceptor to produce methane. The reaction involves a series of enzymes and coenzymes. Understanding the underlying mechanism of methanogen community dynamics and metabolic diversity during solid waste decomposition is critical for methane production and recovery.

Methanogens are the predominant archaea in landfills (Song et al., 2015a) and perform the terminal step of methane conversion (Garrity and Holt, 2001). According to the metabolic precursors consumed, methanogens are classified into three groups as acetoclastic methanogens (acetate), hydrogenotrophic methanogens (H_2_, formate, and CO_2_), and methylotrophic methanogens (methylated compounds) (Garrity and Holt, 2001; Vanwonterghem et al., 2016). Both acetoclastic and hydrogenotrophic methanogens in solid waste have been reported (Barlaz, 1997; Song et al., 2015a; De la Cruz et al., 2021; Zhao et al., 2021), and their abundance varied under different conditions. Few studies have investigated the methanogen community dynamics during solid waste decomposition. Monitoring changes in δ^13^CH_4_ during the mesophilic fermentation of MSW showed that methanogenic metabolism changed with increasing incubation times (Qu et al., 2009). Conversely, automated ribosomal intergenic spacer analysis (ARISA) and fluorescence *in situ* hybridization (FISH) observation showed that the methanogen community is kept stable. Fei et al. (2015) studied the methanogen community changes at the methane production phase and found that *Methanobacteriaceae* was dominant throughout the phase. In contrast, we previously investigated the methanogen community in Chinese landfills and results showed that hydrogenotrophic methanogens *Methanomicrobiales* and/or *Methanobacteriales* were dominant (Song et al., 2015a). This discrepancy may reflect different components in solid waste in various regions. In addition, the study of Qu (Qu et al., 2009) only identified the methanogenesis phase and we only investigated methanogens in limited time points rather than the entire process.

Solid waste decomposition is a typical anaerobic digestion (AD), a multiphase complex biological process (Zhao et al., 2019). Methane production by AD depends on the stability and biological activity efficiency of the reaction. Core parameters such as volatile fatty acids (VFAs), pH, gas composition, and production have been used to indicate the stability of reaction (Boe et al., 2010; Polag et al., 2015). Most parameters, however, have deficiencies due to limited available conditions in responding to the actual condition. A stable carbon isotope shows potential for AD reaction monitoring with high-resolution and fast-responding characteristics (Polag et al., 2015). This technique will benefit from the succession of methanogens and methanogenic pathways during the entire process.

Methanogens’ metabolic function plays an essential role in methane production during solid waste decomposition. Methyl coenzyme M reductase, including two alpha (*mcrA*), two beta (*mcrB*), and two gamma (*mcrG*) subunits, catalyzes heterodisulfide formation between coenzyme M and coenzyme B and subsequent release of methane (Dhillon et al., 2005). Gene *mcrA* has been widely used to investigate methanogens in solid waste (Tang et al., 2016). However, methanogenic pathways and their associated functional genes during solid waste decomposition are not known. The recently developed PICRUSt using 16S rRNA homology analysis has shown great potential for metabolic function prediction (Langille et al., 2013).

In this study, the changes in methanogen community composition and functional genes relevant to methanogenic pathways during solid waste decomposition were investigated in a bioreactor by Illumina MiSeq sequencing and PICRUSt, respectively. The impact of the physiochemical parameters of solid waste and leachate on the methanogen community structure was then assessed by multivariate analysis. Study on the methanogen community dynamics and methanogenic pathways during solid waste decomposition not only provides direct evidence for methane production mechanism but also shows potential for the development of monitoring indicators in AD stability.

## Materials and Methods

### Bioreactor Operation and Solid Waste Sample Collection

Solid waste samples were collected from a long-term-run (265-day) bioreactor (1.0 m height and 0.2 m inner diameter) made of polymethyl methacrylate. The bioreactor was initially compacted with typical Chinese MSW (57.1% food waste, 16.8% plastic, 13.4% paper, 9.1% branches and leaves, and 3.6% fabric and cloth; wet mass) and was recirculated by leachate. The details of reactor construction and operation were previously reported (Liu et al., 2018). Twenty-gram solid waste samples were obtained monthly from three sampling ports with 20 cm inter-distance along the bioreactor. For leachate, 50-ml samples were collected weekly. Analyses of solid waste physiochemical parameters (TN: total nitrogen, nitrate, ammonia, BDM: biodegradable matter, and OM: organic matter) and leachate (COD: chemical oxygen demand, BOD_5_: biological oxygen demand, pH, TN, TP: total phosphorus, nitrate, and ammonia) were carried out as indicated in American Public Health Association (APHA) standard methods (APHA, 1998).

### DNA Extraction

The solid waste samples extracted from three sampling ports were mixed well to represent the entire microbial community. DNA of solid waste samples was extracted by EZNA^®^ Soil DNA Kit (Omega Bio-Tek, Norcross, GA, United States). Extracted DNA quantification and quality assessment followed a previously reported method (Song et al., 2016). DNA extraction was performed in triplicate for each sample.

### Illumina MiSeq Sequencing and Data Treatment

Illumina MiSeq sequencing and data analyses were used to characterize methanogen community composition through primers Arch334F–Arch915R (Raskin et al., 1994). The PCR amplification, purification, and sequencing were described previously (Song et al., 2017). The raw pyrosequencing data were deposited in the NCBI Sequence Read Archive (accession number: SPR118850). Analysis of raw data was carried out as in a previously published procedure (Song et al., 2017). In brief, chimeras were detected by UCHIME. Operational taxonomic units (OTUs) at 97% identity were classified by USEARCH (Edgar, 2010). OTUs were aligned to the SILVA database (version SSU 119) to calculate the bacterial diversity index. Taxonomic classification was performed with the Ribosomal Database Project (RDP) naïve Bayesian rRNA classifier.

### Methanogen Community Composition and Structure Analysis

Hierarchical clustering was used to evaluate the similarity of methanogen community structures. RDA was used to assess the important factors on the methanogen community structure by forward model selection in linear model (Song and Li, 2016). The sum of the eigenvalue score derived from detrended correspondence analysis (DCA) for the methanogen community was 1.3, lower than 3.0. Therefore, RDA was selected in this study. Hierarchical clustering was conducted with the R program (R Core Team, 2013) with the Vegan package (Oksanen et al., 2013). DCA and RDA were performed by CANOCO (ter Braak and Smilauer, 2002).

### Functional Profiling of the Methanogen Community

Methanogenic metabolism functional genes were predicted by PICRUSt (Langille et al., 2013). PICRUSt showed COG and KEGG Ortholog (KO) based on the OTU matrix. EggNOG (evolutionary genealogy of genes: Non-supervised Orthologous Groups^1^) and Kyoto Encyclopedia of Genes and Genomes^2^ were used to annotate the functional genes. Methanogenic pathways were constructed on the KEEG database.

### Statistical Analysis

One-way analysis of variance (ANOVA) was used to test the significance of difference. The value of *p* < 0.05 was significant. Statistical analyses were performed using PAST version 3.0 (Hammer et al., 2001). Data were expressed as the mean ± standard error.

## Results

### Solid Waste Decomposition Phase Separation

A three-stage MSW decomposition model, including aerobic phase (AP), anaerobic acid phase (ACP), and methanogenic phase (MP), was chosen as a phase separation model for solid waste decomposition in this study (Barlaz et al., 1989; Kjeldsen et al., 2002). In AP, microorganisms rapidly consumed the oxygen in MSW and this period usually lasts 1–2 weeks. In ACP, large amounts of carboxylic acids were produced. In MP, carboxylic acids were converted to methane and carbon dioxide. Accordingly, the pH value and BOD_5_/COD ratio in leachate have typical value ranges in three stages, which were defined as the key parameters for the three-stage model (Kjeldsen et al., 2002). In this study, three stages were successfully separated according to the pH value and BOD_5_/COD ratio in leachate as the key parameters in Liu et al. (2018). However, other physiochemical parameters of leachate except the BOD_5_/COD ratio and pH value varied with those in Liu et al. (2018).

**FIGURE 1:**
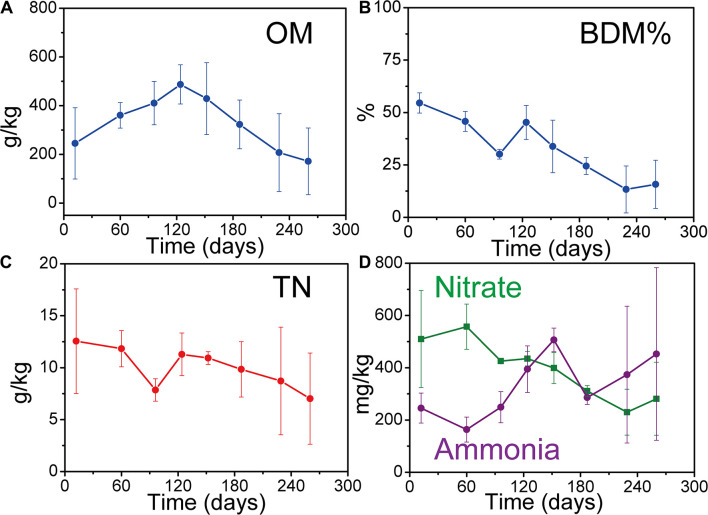
Changes in solid waste key physiochemical parameters during solid waste decomposition. **(A)** Organic matter (OM) of solid waste. **(B)** Percentage of biodegradable matter (BDM %) of solid waste. **(C)** Total nitrogen (TN) of solid waste. **(D)** Nitrate and ammonia of solid waste. Each sample was determined in triplicate (*n* = 3). Error bar is the standard deviation of triplicate determination.

Solid waste physiochemical parameters (TN, nitrate, ammonia, BDM%, and OM) varied with solid waste decomposition (Figure 1). The trends of these variations are consistent with previously reported solid waste decomposition characteristics (Youcai et al., 2000; Kjeldsen et al., 2002). The values of OM, TN, BDM%, and nitrate were lower in the later part of AD than in the beginning. Conversely, the concentrations of ammonia were higher in the later part of AD than in the beginning due to the accumulation (Kjeldsen et al., 2002). In addition, the values of OM peaked at day 120 due to the macromolecule (e.g., cellulose) decomposed into micromolecule (e.g., sugars) (Youcai et al., 2000).

The percentage of BDM decreased from 55 ± 5% in AP to 30 ± 2% in ACP, and further to 13 ± 11% in MP, resulting in 76% BDM loss by microorganism consumption. This significant BDM% loss (*p* = 0.001) indicated that solid waste biodegradation occurred.

### Richness and Diversity of the Methanogen Community

**FIGURE 2:**
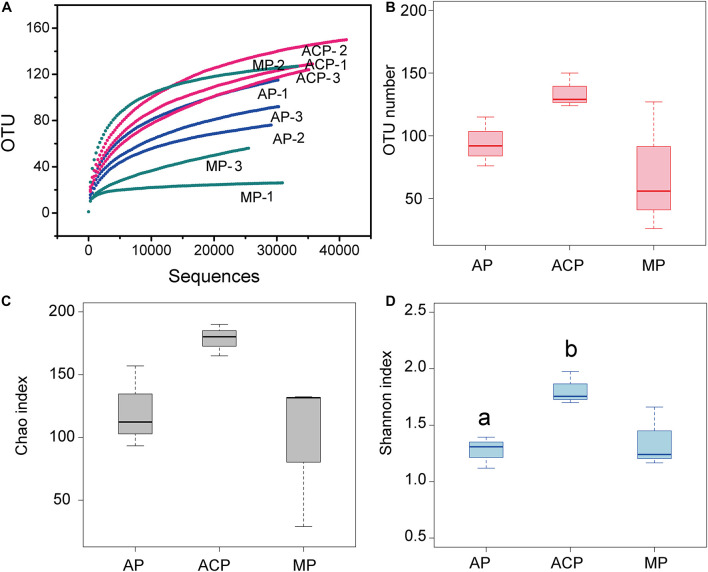
Sequence rarefaction curve and archaeal diversity of solid waste samples. **(A)** Sequence rarefaction curve. **(B)** Observed OTU numbers. **(C)** Chao richness index. **(D)** Shannon diversity index. Each sample was determined in triplicate (*n* = 3). Data were shown as average ± SD. AP: aerobic phase (*n* = 3); ACP, anaerobic acid phase (*n* = 3); MP, methanogenic phase (*n* = 3). Values with different letters were significantly different (*p* < 0.05).

In total, we obtained 320,215 valid sequences with an average length of 526 bp. Each sample had an average of 35,579 ± 4,594 sequence reads. Rarefaction curves showed that all samples reached the plateau phase by 20,000 sequence reads (Figure 2A). Richness (OTUs and Chao 1) and diversity (Shannon) of the methanogen community are shown in Figure 2. The highest richness of the methanogen community in solid waste was ACP, followed by AP and MP. Additionally, ACP has significant (*p* = 0.03) high diversity indicated by the Shannon index than AP.

### Dynamics of Taxonomic Composition at Order Levels

**FIGURE 3:**
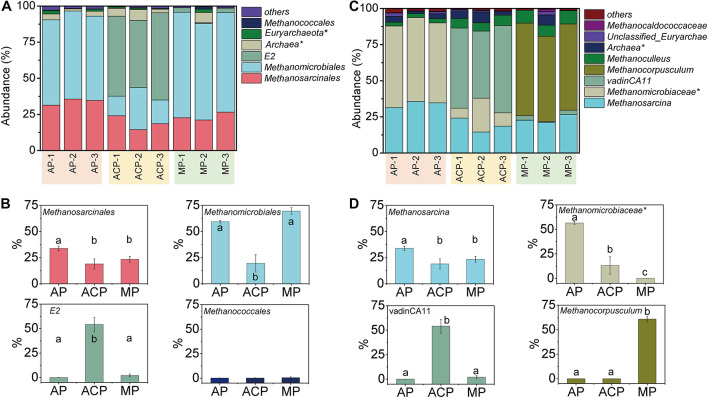
Methanogen community composition in order level **(A)** and genus level **(C)** and main order **(B)** and genus **(D)** changed in solid waste samples during solid waste decomposition. Phylogenetic groups accounting for ≥0.50% of the sequences are summarized in the artificial group “others.” AP, aerobic phase (*n* = 3); ACP, anaerobic acid phase (*n* = 3); MP, methanogenic phase (*n* = 3). Values with the different letters were significantly different (*p* < 0.05).

The orders of methanogen abundant in solid waste decomposition were *Methanomicrobiales*, *Methanosarcinales*, *Methanococcales*, and archaea E2 (Figure 3A). Among them, *Methanomicrobiales*, *Methanosarcinales*, and E2 were the three most dominant groups, accounting for 93.5–95.9% of the total.

These three most dominant groups varied at three phases of solid waste decomposition (Figure 3B). The abundance of *Methanosarcinales* in AP was significantly high than in ACP (*p* = 0.005) and MP (*p* = 0.02). The abundance of *Methanomicrobiales* in AP (*p* = 0.0004) and MP (*p* = 0.0003) was significantly high than in ACP. The abundance of E2 in ACP was significantly high than in AP (*p* = 0.0002) and in MP (*p* = 0.0002). The abundance of *Methanococcales* in AP (2.8 ± 0.2), ACP (6.5 ± 0.7), and MP (8.6 ± 0.8) was relatively low, and the differences were not significant.

### Dynamics of Taxonomic Composition at Genus Level

Genera *Methanosarcina* (affiliated to *Methanosarcinales*), unclassified *Methanomicrobiaceae* (affiliated to *Methanomicrobiales*), vadinCA11 (affiliated to E2), and *Methanocorpusculum* (affiliated to *Methanomicrobiales*) were the dominant genera during solid waste decomposition (Table 1 and Figures 3C,D). The abundance of *Methanosarcina* was significantly high in AP than in ACP (*p* = 0.005) and MP (*p* = 0.02). Similarly, the abundance of unclassified *Methanomicrobiaceae* was significantly high in AP than in ACP (*p* = 0.0002) and in MP (*p* = 0.0002). However, the abundance of *Methanocorpusculum* was significantly higher in MP than in AP (*p* = 0.0002) and in ACP (*p* = 0.0002).

**TABLE 1:** Main methanogen genus during solid waste decomposition phases.

Order	Genus	AP (%)	ACP (%)	MP (%)
*Methanosarcinales*	*Methanosarcina*	33.9^a^ (±2.2)	19.0^b^ (±4.8)	23.6^b^ (±2.9)
E2	vadinCA11	0.03^a^ (±0.06)	54.1^b^ (±7.1)	2.2^a^ (±1.4)
*Methanomicrobiales*	*Methanocorpusculum*	ND	0.02^a^ (±0.02)	61.0^b^ (±2.7)
*Methanomicrobiales*	*Unclassified Methanomicrobiaceae*	56.6^a^ (±1.4)	13.2^b^ (±8.9)	0.02^c^ (±0.01)
*Methanomicrobiales*	*Methanoculleus*	2.8^a^ (±0.2)	6.5^b^ (±0.7)	8.6^c^ (±0.8)

*ND, not detected. Values with the same letters were not significantly different (*p* < 0.05).*

### Dynamics of the Core Operational Taxonomic Units

**FIGURE 4:**
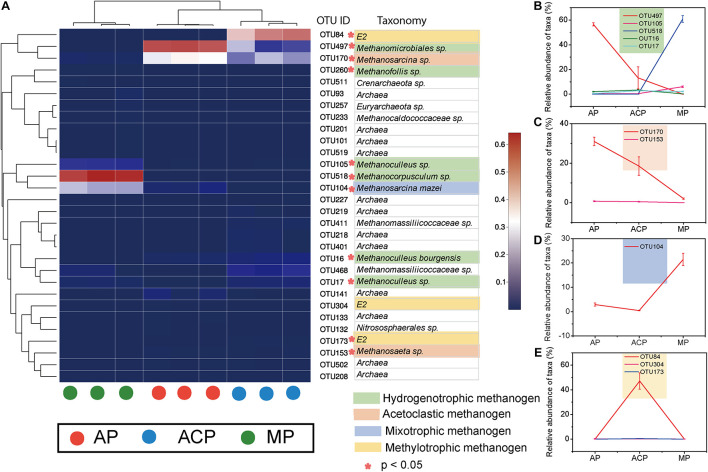
The 30 most abundant OTUs **(A)** and main abundant OTUs **(B–E)** in response to solid waste decomposition. *The difference between the three phases was significant (*p* < 0.05). AP, aerobic phase (*n* = 3); ACP, anaerobic acid phase (*n* = 3); MP, methanogenic phase (*n* = 3).

The 30 top abundant OTUs clearly separated solid waste decomposition into three groups, namely, AP, ACP, and MP, suggesting their core function in shaping methanogen community structure (Figure 4A). A total of 11 core OTUs were significantly (*p* < 0.05) different between AP, ACP, and MP. OTUs with IDs 497, 105, 518, 16, and 17 were assigned to hydrogenotrophic methanogen (Figure 4B). OTUs with IDs 170 and 153 were assigned to acetoclastic methanogen (Figure 4C). OTUs with ID 104 were assigned to mixotrophic methanogen (Figure 4D). OTUs with IDs 84, 304, and 173 were assigned to methylotrophic methanogen (Figure 4E).

### Methanogen Community Structures and Their Linked Impact Factors

**FIGURE 5:**
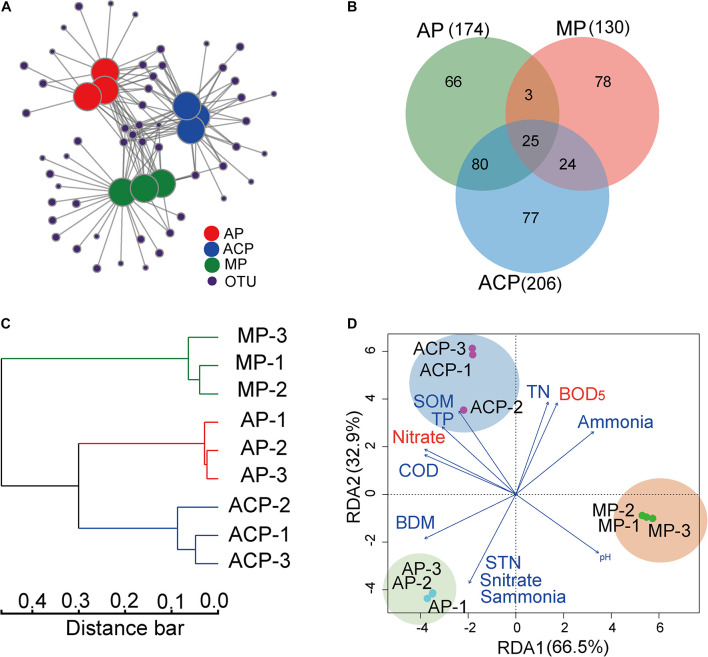
OTU profiles in response to solid waste decomposition phases and the impact of parameter factors on the methanogen community structure based on OTU distribution. **(A)** Network analysis of OTUs in solid waste samples between aerobic phase (AP), anaerobic acid phase (ACP), and methanogenic phase (MP). **(B)** The shared OTUs in solid waste samples among AP, ACP, and MP. **(C)** Hierarchical clustering analysis on bacterial community composition (OTUs level) based on Bray–Curtis distance. **(D)** Redundancy analysis (RDA) of the link between methanogen community structures and physiochemical parameters of leachate and solid waste. The variables significantly influencing methanogen community structure selected by the forward selection procedure were fitted to the ordinations with red color. AP, aerobic phase (*n* = 3); ACP, anaerobic acid phase (*n* = 3); MP, methanogenic phase (*n* = 3). STN, TN of solid waste; Snitrate, nitrate of solid waste; Sammonia, ammonia of solid waste; SOM, organic matter of solid waste.

Network analysis (Figure 5A) and hierarchical clustering analysis (Figure 5C) based on OTU separated the methanogen communities of AP, ACP, and MP. This is consistent with the observed difference between methanogen community composition and core OTUs during solid waste decomposition. AP, ACP, and MP shared 25 common OTUs (Figure 5B). Nine of 11 core OTUs (OTU 83 and 173 excluded) that derived from the top 30 OTUs were also within 25 common OTUs, suggesting their vital role in methanogenesis.

The link among the methanogen community structure and leachate and solid waste physiochemical parameters was analyzed by RDA (Figure 5D). COD, BOD_5_, pH, TN, TP, nitrate, and ammonia of leachate and TN, nitrate, ammonia, biodegradable matter (BDM), and organic matter of solid waste were chosen as environmental variables by forward selection in the RDA model to explain the variation of community. Among them, BOD_5_ (F-ratio, 42.2; *p* = 0.001) and nitrate (F-ratio, 11.7; *p* = 0.01) of leachate showed a significant impact on shaping the methanogen community.

### Predicted Functional Enzyme-Encoding Genes of Methanogenic Pathways

Functional enzyme-encoding genes involved in the three main methanogenic pathways (acetoclastic, hydrogenotrophic, and methylotrophic pathways) during solid waste decomposition were extracted from the KEGG database (Song et al., 2017; Buhlmann et al., 2019).

**FIGURE 6:**
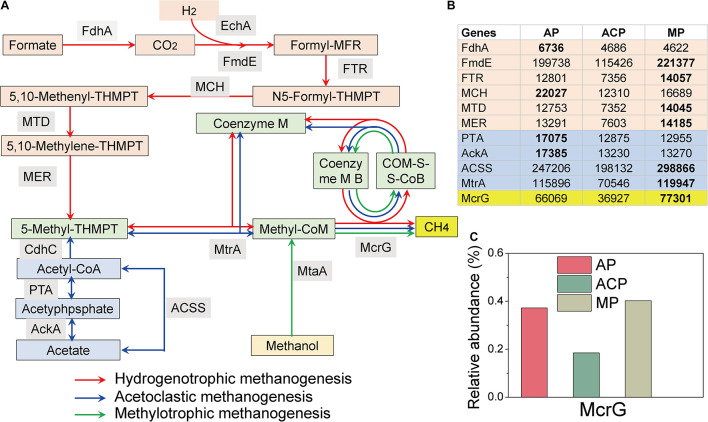
Hit reads of genes involved in the hydrogenotrophic (red line), acetoclastic (blue line), and methylotrophic (green line) methanogenesis pathways during solid waste decomposition **(A)**. **(B)** The hit reads of relevant genes. **(C)** The relative abundance of the core mcrG gene. The methanogenesis pathway figure (Yang et al., 2021) was modified based on Li et al. (2017a). FdhA, glutathione-independent formaldehyde dehydrogenase; EchA, hydrogenase subunit A; FmdE, formylmethanofuran dehydrogenase subunit E; FTR, formylmethanofuran-tetrahydromethanopterin *N*-formyltransferase; MCH, methenyltetrahydromethanopterin cyclohydrolase; MTD, methylenetetrahydromethanopterin dehydrogenase; MER, coenzyme F420-dependent N5, N10-methenyltetrahydroethanopterin reductase; MtrA, tetrahydromethanopterin S-methyltransferase; MtaA, [methyl-Co(III) methanol-specific corrinoid protein]: coenzyme M methyltransferase; McrG, methyl-coenzyme M reductase gamma subunit; AckA, acetate kinase; ACSS, acetyl-CoA synthetase; PTA, phosphate acetyltransferase; HdrA, heterodisulfide reductase subunit; CdhC, acetyl-CoA decarbonylase/synthase complex subunit beta.

In the hydrogenotrophic pathway (Li et al., 2017a), carbon dioxide is reduced to methane *via* a series of intermediate substances (formyl, methylene, and methyl). The methyl group is converted by Coenzyme M to form methyl-CoM, which is reduced to methane through methyl coenzyme M reductase (Mcr) (red line in Figure 6A). In the acetoclastic pathway (Li et al., 2017a), methanogen *Methanosarcina* utilizes the low-affinity acetate kinase (AK)-phosphotransacetylase (PTA) system to convert acetate to acetyl-CoA, whereas *Methanosaeta* uses the high-affinity adenosine monophosphate (AMP)-forming acetyl-CoA synthetase. Acetyl-CoA is converted to a methyl group and subsequently to methane through the key enzymes of Cdh, Mtr, and Mcr (blue line in Figure 6A). In the methylotrophic pathway (Li et al., 2017a), the methylated compounds are converted to methanol-specific corrinoid protein. Methyl-CoM subsequently reduces the protein to methane *via* Mcr reductase (green line in Figure 6A).

The detected enzyme-encoding genes involved in the acetoclastic pathway include acetate kinase (AckA), phosphate acetyltransferase (PTA), acetyl-CoA synthetase (ACSS), and tetrahydromethanopterin *S*-methyltransferase (MtrA) (Figure 6B). MP (298866/119947) had higher hit reads of ACSS and MtrA than AP (247,206/115,896) and MP (198,132/70,546). AP (17385/17075) had higher hit reads of AckA and PTA than ACP (13,230/12,875) and MP (13,270/12,855).

The detected enzyme-encoding genes involved in the hydrogenotrophic pathway include glutathione-independent formaldehyde dehydrogenase (FdhA), formylmethanofuran dehydrogenase subunit E (FmdE), formylmethanofuran-tetrahydromethanopterin *N*-formyltransferase (FTR), methenyltetrahydromethanopterin cyclohydrolase (MCH), methylenetetrahydromethanopterin dehydrogenase (MTD), coenzyme F420-dependent N5, and N10-methenyltetrahydroethanopterin reductase (MER) (Figure 6B). Except FdhA, the rest of the five genes had higher abundance in MP than those in AP and ACP.

The enzyme-encoding gene involved in the methylotrophic pathway is [methyl-Co(III) methanol-specific corrinoid protein]: coenzyme M methyltransferase (MtaA) (Figure 6B), which is not detected in this study. The abundance of methylotrophic methanogen E2 in ACP might express other functions than methylotrophic methanogenesis.

In addition, the percentage and hit reads of McrG were lower in ACP (0.18%/36,927) than in AP (0.38%/66,069) and MP (0.40%/77,301) (Figure 6C), further demonstrating that methane production was inhibited in ACP.

## Discussion

Landfills are the most commonly used site for MSW disposal. Large amounts of organic matter in landfills undergo a series of decomposition processes. The decomposition process has been modeled, and the three-stage (AP, ACP, and MP) MSW decomposition model is one of the most classic phase separation models (Barlaz et al., 1989; Kjeldsen et al., 2002). In this study, these three stages were successfully separated according to the physicochemical parameters of leachate (Liu et al., 2018). In addition, the values of OM, TN, BDM%, and nitrate decreased along the decomposition. There was 76% BDM lost by microorganism consumption. The combination of these physicochemical parameters’ variation showed that solid waste undergoes decomposition.

Landfills harbor highly diverse and dynamic microbial communities. Within them, the methanogen community plays a vital role in methane production. Obviously, the methanogen community responds to the variation in the decomposition condition. In this study, the methanogen community richness and diversity differed at three solid waste decomposition phases (e.g., ACP had the highest richness of methanogen community). Taxonomically, *Methanomicrobiales*, *Methanosarcinales*, and E2 were the three most dominant groups. Domination of *Methanomicrobiales* and *Methanosarcinales* was also detected in other studies on solid waste decomposition (Bareither et al., 2013; Fei et al., 2015; Song et al., 2015a), whereas the dominance of E2 was for the first time detected. This is probably due to most of the previous studies investigating the methanogen community in specific time points rather than the whole solid waste decomposition process. These three most dominant groups varied at the three phases of solid waste decomposition. *Methanomicrobiales* and *Methanosarcinales* were initially the dominant populations in AP, E2 became the dominant group in ACP, and *Methanomicrobiales* and *Methanosarcinales* dominated in MP again. High VFAs produced by high food waste (57.1%, mass percent) in this study probably cause the decrease in *Methanomicrobiales* and *Methanosarcinales* in ACP due to VFA inhabitation (Song et al., 2015a). E2 (affiliated to class *Thermoplasmata*) was abundant in ACP as well as in other AD (Godon et al., 1997; Leclerc et al., 2004), owing to their resistance ability on acidophilic and thermophilic conditions. *Thermoplasmata* in bovine rumen has been proved to be methylotrophic methanogens by metatranscriptomic analysis (Poulsen et al., 2013). Dominant methylotrophic methanogen E2 was detected in ACP of solid waste decomposition, highlighting the critical role of this group in acidogenesis. In addition, the methanogen community in solid waste differs from that in landfill leachate (Yang et al., 2021). For landfill leachate, E2 (32.2%) and *Methanomicrobiales* (35.3%) dominated in AP, E2 predominated in ACP (98.7%), and *Methanomicrobiales* predominated in MP (92.4%). The difference might be the microorganisms’ different dispersal in solid and liquid phases.

Studies on the methanogen community dynamics during methane production showed that hydrogenotrophic methanogens [*Methanomicrobiales* (Bareither et al., 2013) or *Methanobacteriaceae* (Fei et al., 2015)] were dominant throughout the phase, which is consistent with this study that hydrogenotrophic methanogen *Methanomicrobiales* was dominant in MP. However, our study showed that the methanogen community composition changed during solid waste decomposition. Two reasons might contribute to the difference. First, these two studies sampled in-place solid waste for approximately 3–4 months (Bareither et al., 2013) and 2–3 years (Fei et al., 2015), respectively. Therefore, the aerobic phase is absent due to AP occurring in a short term (usually 1–2 weeks) (Kjeldsen et al., 2002; Song et al., 2015b). Second, the abundance of food waste in tested solid waste was low (1.1%) or zero, much lower than that in this study (57.1%). In this study, AP was separated during solid waste decomposition based on previously published results (Kjeldsen et al., 2002) and under *in situ* bioreactor operation conditions. Abundances of *Methanosarcinales* and *Methanomicrobiales* were detected in solid waste samples of AP and MP. Acidic and thermal conditions in ACP favor the growth of archaea E2 (Godon et al., 1997; Leclerc et al., 2004), likely leading to abundant E2 occurring in solid waste.

Methanogen genera varied according to the variation of the methanogen order. Although methanogen *Methanomicrobiales* were dominant in AP and MP, their affiliated genera changed, showing that unclassified *Methanomicrobiaceae* was abundant in AP and *Methanocorpusculum* was dominant in MP. Hydrogenotrophic methanogens *Methanomicrobiaceae* and *Methanocorpusculum* have been reported in AD (Carballa et al., 2015) and molasses wastewater anaerobic reactor (McHugh et al., 2004) due to their tolerance to environmental stress. Acetoclastic methanogens such as *Methanosarcina* sp. have also been reported in AD (McHugh et al., 2003; Laloui-Carpentier et al., 2006; Langille et al., 2013), indicating that acetoclastic methanogenesis pathways extensively exist in AD. Genus vadinCA11 (affiliated to E2) was also abundant in AD (Godon et al., 1997; Leclerc et al., 2004), owing to its acidophilic and thermophilic adaption characters.

The 30 core top abundant OTUs clearly separated solid waste decomposition into three groups AP, ACP, and MP. At the same time, network analysis and hierarchical clustering analysis separated the methanogen community into three groups AP, ACP, and MP. The combination of these analyses showed that methanogen composition dramatically varied during solid waste and had unique patterns at each phase of decomposition.

RDA analysis showed that BOD_5_ and nitrate of leachate were the significant impact factors in shaping the methanogen community. BOD_5_ contains methane metabolic precursors, which are used with methanogen to produce methane. Nitrate has been found as the important factor associated with the methanogen community in landfill (Song et al., 2015a). However, the underlying mechanism of nitrate in driving the methanogen community is unknown. The link between methanogen community structure and important parameters (BOD_5_ and nitrate) reflected that methanogen community variation accords with changes in physiochemical parameters.

Functional enzyme-encoding genes involved in acetoclastic, hydrogenotrophic, and methylotrophic pathways during solid waste decomposition were determined. For all detected genes in the acetoclastic pathway, AP and MP had higher hit reads than ACP, suggesting that the acetoclastic pathway was inhibited in acidogenesis. The high stress of acidogenesis such as high VFA inhabitation on acetoclastic methanogen has been observed in AD (Karakashev et al., 2006). Except FdhA, the rest of the five genes (FmdE, FTR, MCH, MTD, MER) of the hydrogenotrophic pathway were more abundant in MP than those in AP and ACP. This observation accords with the function of MP that methane production mainly occurs at this phase. The combination of the abundant genes’ involvement in the hydrogenotrophic pathway and our observation on dominant hydrogenotrophic methanogens in Chinese landfills (Song et al., 2015a) indicates that hydrogenotrophic methanogen and its methanogenic pathways significantly contribute to the methane production in solid waste decomposition. In theory, acetate and hydrogen contribute 67% and 33% of total methanogenesis, respectively (Conrad, 1999). However, increasing evidence showed that hydrogenotrophic methanogenesis contributes more CH_4_ production than previously considered (Karakashev et al., 2006; Krakat et al., 2010; Nettmann et al., 2010), likely due to the higher stress tolerance ability of hydrogenotrophic methanogen than acetoclastic methanogen (Song et al., 2015a). The MtaA gene of the methylotrophic pathway was not detected in this study. The abundance of methylotrophic methanogen E2 in ACP might express other functions than methylotrophic methanogenesis. In response to the variation of the metabolic pathways and the McrG genes, involvement in methane production was inhibited in ACP.

In this study, methanogenesis functional enzyme-encoding genes were predicted based on 16S rRNA homology analysis, showing the potential for methanogenesis. The detected abundant hydrogenotrophic and acetoclastic methanogenesis functional genes in MP and AP were in response to the abundance of *Methanomicrobiales* and *Methanosarcinales*. Further studies based on RNA and protein high-throughput analysis are necessary to investigate the activated functions.

## Conclusion

Methanogen community composition varied at AP, ACP, and MP, which is driven by BOD_5_ and nitrate. *Methanomicrobiales* and *Methanosarcinales* were dominant in AP and MP, while E2 was abundant in ACP. The main methanogen orders and genera were significantly different during solid waste decomposition. The detected abundant hydrogenotrophic and acetoclastic methanogenesis functional genes in MP and AP are in response to the abundance of *Methanomicrobiales* and *Methanosarcinales*. The findings provide information on the methanogen community and methanogenesis functional genes in methane production in solid waste decomposition.

## Data Availability Statement

The original contributions presented in the study are included in the article/supplementary material. Further inquiries can be directed to the corresponding author/s.

## Author Contributions

SY, LS, and XP conceived of and designed the research. SY, LL, and RZ conducted the research. SY, LL, XP, and LS contributed to discussions and suggestions. SY analyzed the data. SY and LS wrote the manuscript. All authors read and approved the manuscript.

## Conflict of Interest

The authors declare that the research was conducted in the absence of any commercial or financial relationships that could be construed as a potential conflict of interest.

## Publisher’s Note

All claims expressed in this article are solely those of the authors and do not necessarily represent those of their affiliated organizations, or those of the publisher, the editors and the reviewers. Any product that may be evaluated in this article, or claim that may be made by its manufacturer, is not guaranteed or endorsed by the publisher.

## Footnotes

1
http://eggnog.embl.de/

2
http://www.genome.jp/kegg/
